# Family influence and psychiatric care: Physical treatments in Devon mental hospitals, c. 1920 to the 1970s^☆^

**DOI:** 10.1016/j.endeavour.2013.06.005

**Published:** 2013-09

**Authors:** Nicole Baur

**Affiliations:** University of Exeter, Centre for Medical History, Exeter, Devon EX4 4RJ, United Kingdom

## Abstract

‘What is it that appears to make the mentally ill so vulnerable to therapeutic experimentation?’^1^ One commentator wrote in the 1990s, regarding mental hospitals as repressive, coercive and custodial institutions where medical staff subjected patients to orgies of experimentation. A careful study of surviving documents of the Devon County Lunatic Asylum (DCLA), however, paints a different picture. Rather than medical staff, patients’ relatives and the wider community exercised a considerable influence over a patient's hospital admission and discharge, rendering the therapeutic regime in the middle of the 20th century the result of intense negotiations between the hospital and third parties.

## Highlights

• In the 20th century a variety of short-lived treatments were trialled at the DCLA.
• Therapeutic regimes depended on institutional, legislative and social factors.
• The 20th century witnessed an increase in the medicalisation of mental illness.
• The 1930 Mental Treatment Act granted patients slightly greater agency.
• Deinstitutionalisation did not eliminate coercion in psychiatric treatment.

## Patient 254

In 1924, female patient 254^2^ was admitted to the Devon County Lunatic Asylum (DCLA).^3^ Single, in her mid-thirties, and employed as a nursemaid, she was suffering from her first attack of ‘recent mania’ that had started one week prior to admission. Her mother, listed as next-of-kin, told the Relieving Officer that the patient had a habit of purchasing ‘articles in several shops that are not necessary and which she is unable to afford. She stops cars in the streets and asks the occupants to give her rides. She uses abusive language and also obscene, which is not usual. She has struck me on several occasions,’ reported the mother, ‘and broken many pictures’. The patient's sister further explained that the patient had suffered a sunstroke around one year prior to admission, ‘and since then has been very excitable and strange at mense [….] She has never been quite herself since’. Based on these observations the medical practitioner who examined patient 254 prior to admission declared her ‘a person of unsound mind’. Disregarding the relatives’ suggestive aetiology, the doctor considered the illness to be hereditary – probably because a cousin of the patient had been ‘afflicted with insanity’.

The patient was discharged ‘to the responsibility of friends’ after about two months, only to be readmitted two weeks later. Until her death when hospitalised in 1969, she would have multiple voluntary and compulsory admissions. In line with the increasing medicalisation of mental illness during the 20th century, she was treated with a variety of physical therapies, many of which were beyond her control, as consent was sought from relatives rather than herself.

Patient choice and empowerment have for the past decade been key phrases in an ambitious plan by the British government to allow patients suffering from mental disorders to embark on treatments of their own choice. Until far into the 20th century constraints by the law, the organisation of institutions as well as the therapies available limited patient agency. This was further exacerbated by the brevity of information provided to patients and their kin about treatments. Patient 254 was admitted for the first time at the start of major changes in the therapeutic regime and legislative framework in mental hospitals. Although a tentative step towards greater patient agency, the reforms did little to reduce the influence of relatives who, in their attempt to help the patient, often exercised considerable control over a patient's admission, treatment and release from hospital.

Using the context of physical therapies, this article seeks to construct a greater understanding of the relationship between lay people (i.e. patients and their relatives) and the mental institution during the period before the empowerment of patients was placed high on the governmental agenda with the start of the new millennium. This paper focuses on the 1920s to the 1970s, but a brief excursion to the 19th century will be necessary to understand the legislative and organisational framework in which mental hospitals operated during the middle decades of the 20th century.

## Sources

The research supporting this paper has exploited a diverse range of archival sources, capturing the views of medical experts and lay people alike. Extracting the contributions made by relatives of hospitalised patients from sources available to historians is notoriously difficult. Little can be gained from case notes – however florid they may be – but admission papers, in particular Reception Orders, and surviving correspondence with Poor Law Officers have provided useful information concerning the committal and discharge of patients. How relatives penetrate the power structure of the mental hospital, getting actively involved in a patient's treatment, is even more difficult to reconstruct. Here, correspondence between relatives and the Medical Superintendent has proven useful, as it casts light on the relationship between different agencies involved in the care of the mentally unwell. Such information has been supplemented with Medical Superintendents’ reports and interviews with former staff.

Research presented in this paper complements existing institutional monographs, but introduces a new perspective by focusing on the 20th century, a relatively calm period at the DCLA following its celebrity status under its first Medical Superintendent John Charles Bucknill (1817–1897). Forsythe et al. have examined the shifting nuances of administrative power at the DCLA, and this paper builds on this approach by emphasising the social context, thereby examining treatment from a fresh perspective.^4^ Specifically it argues that control and patient choice need to be understood in relation to the technologies of therapies available, the institutional framework and strategy of care and the scope for patients and their advocates to influence treatments. In doing so, the article focuses on the nature and quality of relationships between lay people and mental hospitals rather than labelling the Medical Superintendent as someone who ‘controls all […] threads’.^5^

## Past readings of the history of psychiatry

The 19th century witnessed the first tentative developments towards a movement labelled by social scientists the ‘medicalisation’ of mental illness, i.e. the transformation of authority over the mentally ill and their treatment from the legal to the medical profession.^6^ Mental hospitals have often been cited as key examples of this transition and consequently the relationship between society and mental hospitals has largely been explored in terms of the dichotomy of humanitarianism versus social control. 19th and early 20th century medical professionals viewed mental disorders largely as biologically rooted and examined the history of mental health treatment in an attempt to establish and legitimise their discipline within mainstream medicine.^7^ Guided by the limited expectations of the time, they often uncritically exaggerated the institution's progressive, therapeutic character, as evidenced by the Devon Superintendent's reports dating from the late 19th and early 20th centuries. Some revisionists, including Grob^8^ and Shorter,^9^ opined, albeit less enthusiastically than the Devon Superintendents, that the institution's good intentions outweighed its controlling function. Grob, for example, acknowledged the coercive element inherent in institutional treatment resulting from diminished autonomy and personal freedom, but admitted to the emergence of a social demand for institutional treatment for the mentally ill, particularly from families residing in close proximity to such institutions.^10^ By attributing the move from therapeutic to custodial care to the increasing asylum population, Grob's account distanced itself from social control theorists who condemned institutions as repressive, coercive and custodial environments where an array of treatments were enthusiastically welcomed and liberally trialled with the aim of normalising or disciplining the socially deviant. The most notable scholars disputing the allegedly linear progress from the barbaric towards humanitarian treatment of the mentally unwell are Foucault,^11^ Goffman,^12^ and Rothman^13^ whose works imply that medicalisation and institutionalisation merely replaced other social institutions. Foucault described how as early as the 17th century the mentally ill were regarded as less acceptable human beings and consequently he regarded psychiatry as an instrument of social control derived from society's desire to control ‘the deviant’. As the mentally disturbed patient does not conform with social norms, society feels invited to impose outer will on them. Social norms are changeable and social deviance once regarded legal can be redefined as mental illness. Thus, definitions and responses to mental illness are very much products of their time. According to Foucault the ‘great confinement’ of the mentally ill was a direct result of such changes in perception. They were related to socio-economical developments, and both Rothman and Scull saw capitalist movements at the bottom of the growing institutionalisation.^14^ For Rothman, asylums were not dissimilar to prisons in their function to restore social order by incarcerating the deviant, whereas Scull blamed industrial capitalism with its strong emphasis on productivity for the rising asylum admissions. Social control theorists also argued that isolating the mentally ill in asylums provided ultimate authority and control to medical staff – a point this paper vehemently refutes. Although institutionalisation (and medicalisation) is ultimately about control and power, this movement was not the result of ‘medical imperialism’,^15^ but rather intense social and political interactions, and it will be shown throughout the article that patients and their relatives requested as well as resisted particular treatments.

Following Foucault, social control theorists claimed that 19th century asylum doctors abused this power for their own professional advance by imposing a scientific model on behavioural disorders.^16^ Thus, their accounts stand in stark contrast to Shorter's^17^ who viewed the origin of the profession in the mission to treat mental illness. Scull's earlier writings^18^ are similarly critical of institutions, but his more recent research^19^ acknowledges that psychiatry could only gain control over insanity owing to a general trend in the social control practices of modern societies aiming to remove the deviant and unproductive from their midst. Such well-known models of control have since been sharply criticised and revised owing to their treatment of the patient as a passive instrument in the hands of the psychiatrist.

## A new perspective

Detailed institutional studies across Britain,^20^ continental Europe^21^ and the United States^22^ have modified the once-dominant picture of Foucault's ‘total institution’.^23^ Recognising the deficiencies of both the humanitarian and the social control approaches, they examined the relationship between the institution and third party agency, placing patients’ families, friends and the wider community centre-stage. Such research recognises that the transformation of a person into a mental patient is a socially structured process rather than the mere outcome of mental illness or the automatic result of a professional opinion, as key decisions about committal were made in the community through families alerting medical authorities to a relative's mental suffering.^24^ Focusing on treatments provided within the walls of the Devon institution, this paper illustrates that families turned into increasingly powerful agents in the therapeutic regime by critically assessing and sometimes undermining the medical authorities. Foucault's as well as Scull's provocative accounts of the growth of asylums cursorily attributed committals to families’ increasing intolerance and unwillingness to look after the insane. Scull in particular suggested that the emerging market-oriented society weakened family bonds and used asylums to rid themselves of their insane family members. Both, however, failed to study family involvement in detail and since then numerous studies have found no evidence that families were unwilling to look after the insane if they were in a position to do so.^25^ Instead, a shift in tolerance of the deviant behaviour has been identified as a critical factor in the admission process,^26^ and Tomes^27^ regarded the institution as filling a social need in the absence of a welfare state by removing the burden of care from the families. It is now generally accepted that the expansion of asylums was driven by complex interactions guided by growing needs from society, power of authorities, and a greater willingness of doctors to specialise in mental illness.^28^ The negotiations between families, Poor Law Officials and doctors in the placement and release of various sub-groups of patients in the 19th century have been studied in detail.^29^ Over the past thirty years, family involvement in treating people with mental illness has become an area of increasing interest, intensified by deinstutionalisation, the acknowledgement of the impact of mental illness on families, and concerns about the role of families in the aetiology of mental illness. It is therefore surprising that little has been done for the earlier decades of the 20th century, as research on the DCLA revealed that this period did not only witness the introduction of many, often hazardous treatments, but also a move towards greater third party agency.

## Devon enters the asylum era

**Figure 1:**
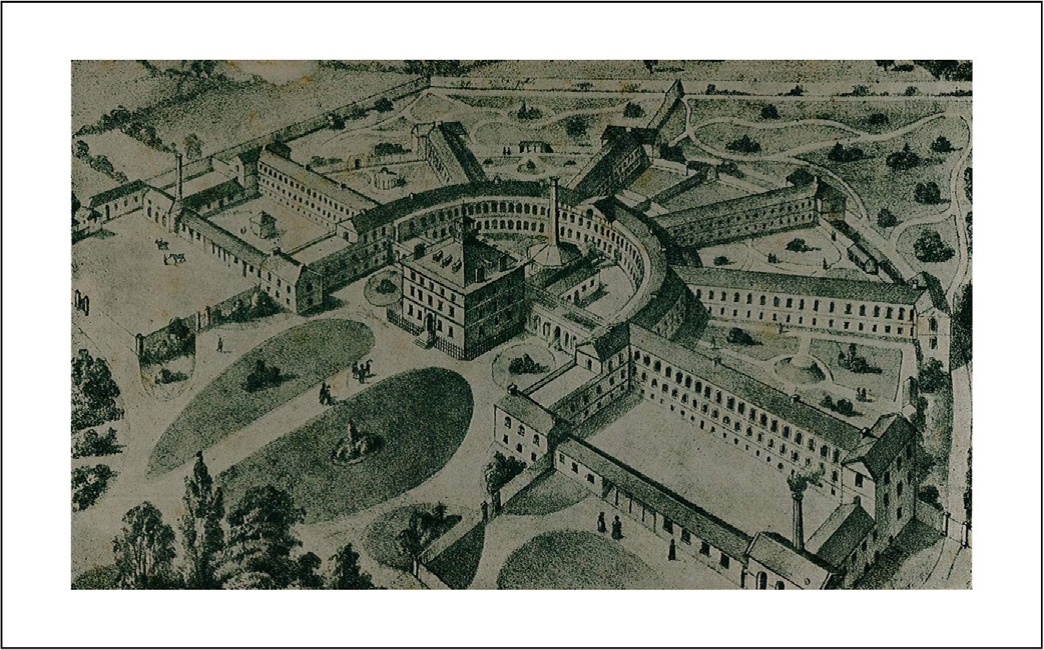
The Devon County Lunatic Asylum, 1845.

The impetus for the construction of the DCLA in the early 1840s arose as much from humanitarianism – triggered by the shocking ill-treatment of many insane by their relatives^30^ and in workhouses – as from the fear created by the apparently growing number of the mentally ill.^31^ In Devon the number of diagnosed insane had risen from 195 in 1828 to 1438 in 1871,^32^ and the DCLA provided the communities with an additional option of dealing with them (Figure 1). Much later revisionists would claim that the mere existence of asylums inflated these numbers. Asylums were places, they argued, to detain the socially unwanted, economically unproductive^33^ and ‘intolerable’ family members,^34^ an argument this paper, in line with many other publications, refutes.

The Medical Superintendent had little influence on who was admitted to his institution, indeed often merely confirmed lay diagnoses.^35^ Thus, Bucknill and his successors were helpless onlookers when the DCLA, in accordance with other mental institutions across the country, quickly filled up. Many long-stay patients were incurable^36^ and not necessarily in need of psychiatric care,^37^ but workhouses had adopted a policy of ridding themselves of the elderly infirm through hospitalisation,^38^ a trend that would continue far into the 20th century. Although ‘certification’ introduced under the 1890 Lunacy Act reduced the control of relatives over the committal process, the Mental Treatment Act (MTA) of 1930, which actively encouraged voluntary admissions, had a profound effect upon the relationship between lay people and mental hospitals. In theory, this form of admission gave greater power to patients to seek treatment, choose from a variety of treatment options, and hence avoid the need – and stigma – of certification (supposedly reserved for patients who could or would not consent to hospitalisation). In reality, however, relatives continued to figure prominently in the admission of patients, embellishing the conditions in the DCLA,^39^ downplaying the duration of hospitalisation,^40^ or invoking the threat of certification. By 1944, with an increase in admissions and serious staff shortages, the Medical Superintendent noted that ‘special treatments which require great attention on the part of the medical and nursing staff’ were beginning to suffer. These treatments, he noted, were still being carried out ‘but not to the extent we would like if the staff were sufficient in numbers’.^41^

The MTA also affected the discharge of patients. Voluntary patients could depart from the hospital after providing 72 h notice. Prior to 1930 the discharge of certified patients required positive action on behalf of the Medical Superintendent, who had to declare them ‘recovered’ and present the case before the Visiting Committee.^42^ This was a considerable responsibility, as the doctor had to anticipate a patient's future behaviour. In spite of this, discharge rates for the DCLA show that the hospital was by no means a ‘dumping ground’ for unwanted relatives in the 19th century – a striking similarity to MacKenzie's findings of the Ticehurst Asylum.^43^ It is, however, not surprising that frequently family members were the driving forces in their relatives’ release.^44^ While emotional and financial aspects, limitations of space as well as the ability to supervise the patient had influenced discharge decisions before, the advent of new treatments in the 1930s instilled in relatives the hope of cure. Some subsequently experimented with the hospital – if their family member did not progress as expected, they would seek their release against medical advice and embark on one of the other options available.

Not all Medical Superintendents were fully satisfied with the new system. Although the opportunity of early treatment and hence increasing chances of recovery should have boosted the reputation of psychiatry, some doctors felt that the possibility of patients refusing treatment or discharging themselves diminished their control.^45^ Patients leaving hospital against medical advice impacted financially on the hospital. It was also feared that patients undergoing malaria treatment might pose a risk to the general public. The 1930 MTA therefore called for unprecedented negotiations between the Medical Superintendent, the patients’ relatives, and also with patients themselves.

## Early treatments

**Figure 2:**
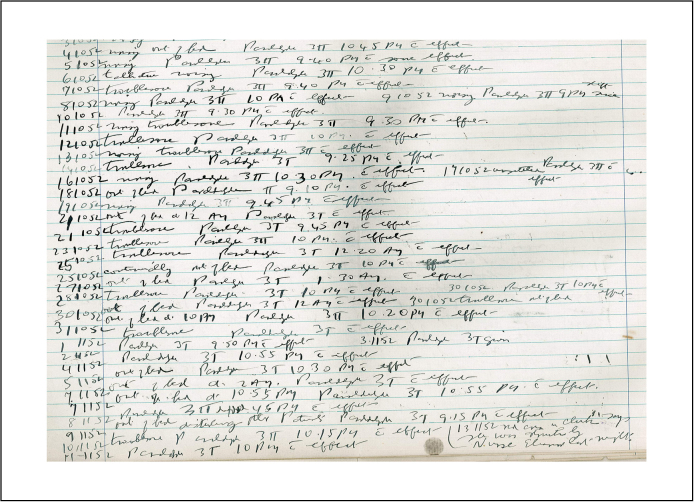
Excerpt of a patient's case notes, which testifies to the administration of paraldehyde whenever the patient became ‘troublesome’.

Patients’ treatment options depended on a variety of institutional, political and social factors.^46^ Initially, therapy was fully controlled by the Medical Superintendent. Bucknill followed a regimen of ‘conciliatory and gentle management, perfect freedom from mechanical restraints and occupation and employment’,^47^ rewarding good behaviour with greater access to the hospital grounds. Under his leadership the DCLA grew into a model institution, although several aspects of this moral treatment have been criticised by revisionists and contemporaries alike. Moral treatment worked by emphasising discipline and a strictly regulated daily routine, a system Foucault labelled a ‘gigantic moral imprisonment’.^48^ Similarly, Goffman^49^ and Rothman^50^ suggested that moral treatment merely replaced physical chains with social restraints. Such broad-brush generalisations resulted from the failure to engage with individual patient files. As will be demonstrated throughout this paper, treatments were administered selectively with the patient's benefit in mind. This is not to suggest that control was entirely absent. A system of non-restraint required patient co-operation as well as close supervision. Bucknill was by no means averse to making patients amenable to this new approach through the application of medication, bleeding and purging,^51^ and if required, punishment through prolonged baths or seclusion.^52^ Moral treatment continued after Bucknill's leave, but was increasingly compromised by staff shortages and the great influx of patients. Conditions sharply deteriorated, the Medical Superintendent's role became increasingly managerial, and patients were ‘to a much greater extent […] slotted into a fixed environment rather than a social context being created for the individual’.^53^ Bucknill had trialled innovations, such as boarding-out for reconvalescing patients adopted from Gheel, but from the 1870s the DCLA, in line with other institutions, increasingly ‘[relied] on pharmacological interventions’.^54^ Morphine, hyoscyamine and hyoscine – often combined with atropine – could be turned into very powerful sleeping draughts for severely disturbed patients. This frequently concerned patients whose noisy and demanding behaviour disturbed the ward routine. Maybe because of its foul odour various Devon staff vividly remember paraldehyde. It was commonly used as sedative from 1882 and although the more modern barbiturates confined it to the back of the medicine cabinet, it was still used in the second half of the 20th century as ‘the great standby’^55^ to calm extremely agitated patients (Figure 2).

At that time patients and relatives had very little influence over the therapeutic regime – apart from removing the patient against medical advice – and consent for treatment was not required. During the entire time period under consideration in this article, sedatives, as any other treatment, could (and would) be applied with force, if necessary^56^ – for punitive purposes^57^ and to improve behaviour on the wards,^58^ thereby alleviating nursing strain. Grob's research confirms a similar situation in the context of the 19th century American asylums, where bizarre patient behaviour resulted in staff applying therapeutic interventions ‘to react to inmate initiative rather than the other way around’.^59^

## The controversy about medical research

Sedatives were also the impetus for medical research at the DCLA, as their use and efficacy began to be debated.^60^ Thus, starting in the late 19th century and increasingly with the emergence of physical treatments in the 20th century, Devon secured its place on the ‘research map’, particularly as the MTA actively encouraged research into mental illness.^61^ Soon, the DCLA Superintendents were delighted with the perceived progress in the cure of mental disturbances in the 1920s and 1930s, as Medical Superintendent Allan points out ‘with the more enlightened treatment of patients, many were the new departures in the years between the wars’.^62^ The following sections illustrate that the new physical treatments and their alleged successes hardly deserved the term ‘enlightened’. Not only did doctors at the time not understand how they worked, but they were carried out at a time when scientific evidence for the efficacy of treatments was lacking and ethical safeguards had yet to be established – in general medicine as well as in psychiatry. By today's standards, the credence of many studies must be regarded as compromised through a selective choice of the study group (often dictated by availability). Furthermore, the efficacy of the new treatments was evaluated by the same physicians who administered them,^63^ the application of a variety of study methods meant that results were not comparable, and findings were usually published long before any long-term effects could be assessed, while deaths during such trials were frequently ignored or put down to pre-existing conditions.^64^ The circulation of such positive results, however, led to widespread acceptance of the new treatments, as did a doctor's reputation in some other area. Egas Moniz's leucotomies, for instance, owed much to his international reputation for having developed cerebral angiography.

The role of patients in these experiments has been heavily debated, usually portraying the patient as the helpless victim.^65^ Contrary to such portraits, evidence from the DCLA illustrates that many patients and relatives welcomed the new treatments, as they often provided the only glimpse of hope for a cure.^66^ A clear shift of control over therapies can be seen in Devon in the 20th century with relatives frequently demanding a certain treatment they had heard or read about – often not through the recommendation of a medical expert but informal channels of information and the emerging mass media. If not cure, then at least improvement was expected from a patient's hospitalisation. If such benefits did not materialise, relatives were quick to remove a patient or demand their transfer to a different – sometimes private – institution.^67^ It is noteworthy though that during the first half of the 20th century, when mental health legislation provided greater freedom for patients in terms of decision-making, treatment options were often compromised by staff shortages and building restrictions. Several financial bids by the DCLA management to expand and modernise the hospital were rejected throughout the late 19th and early 20th centuries. Therefore, all new treatments had to be accommodated within the existing facilities – often with a greater choice for voluntary than for certified patients, if treatments had to be rationed, as it was believed that ‘the more attention and ‘active treatment’ [the voluntary patients] receive, the smaller will be the number of cases who wish to leave before ‘recommended”.^68^ The case notes, however, give little credence to this claim.

The second part of this paper describes the most important physical therapies applied in the DCLA during the 20th century. It will be shown that although doctors were often fully aware of the short lives of their new ‘cures’, the frustration of being unable to help, ongoing professional struggles between psychiatrists and neurologists over the cause and consequently the most beneficial treatment for the mentally ill, combined with a requirement for institutions to reduce their costs and upcoming media influence^69^ resulted in the frequent acceptance of some of the most radical somatic treatments in psychiatry. Such treatments were also easier to implement than psychological approaches: ‘to use the patient's assets is a more difficult problem than using something under our control’.^70^ The article will also illustrate that through many of these treatments doctors and family members were able to exercise considerable control over the hospitalised individual. As has been pointed out before, Foucault and other social control theorists equalled institutionalisation and the treatment provided to the mentally ill in the 19th and early 20th centuries with coercion. This, however, proved too simple an explanation. Similar to 19th century moral treatment, all physical therapies have in common the aim to change a patient's personality in a way to restore their ability of being a fully functioning member of society. Such changes were also in the interest of patients’ relatives whose trust in the new treatments seemed unconditional. While this means that an element of control is inherent in any physical psychiatric treatment, the surviving documents provide extremely little evidence of using the new treatments merely as instruments of social control. In the second half of the 20th century, deinstitutionalisation and community-based treatments were introduced in an attempt to reduce outside control over the mentally ill and increase patient agency. However, although they may be more inclusive, it has been found that treatment in the community is not necessarily less coercive,^71^ and in the UK the legitimacy of compulsory community treatment orders, introduced in the 1990s, have recently been much debated.^72^

## Prolonged narcosis

**Figure 3:**
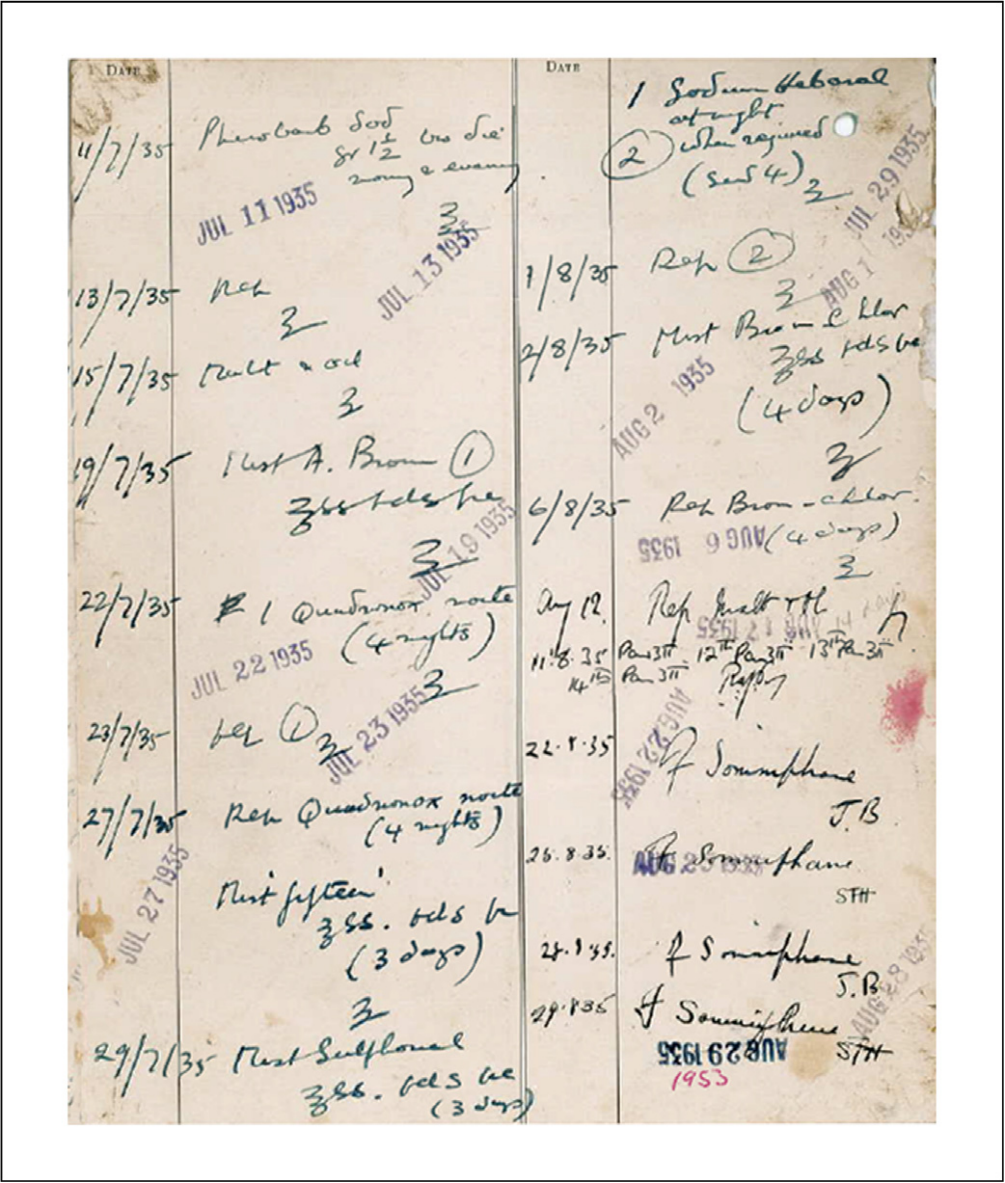
Patient's treatment card, showing prescriptions for somnifaine and other sleep-inducing medication.

Prolonged narcosis with somnifaine to counteract brain overstimulation was introduced in Switzerland in 1921 by Jakob Kläsi. It was the first sedative applied in the DCLA with a true curative function in mind, as opposed to the previous use of sedatives to control patients’ behaviour on the wards. Somnifaine, a strong soporific, was erroneously thought to be much safer than the earlier sleep-inducing drugs,^73^ and case files attest to its extensive use in Devon from 1934. After an injection of 2 cc somnifaine patients slept for approximately five hours, receiving two to three injections daily on several consecutive days (Figure 3).

Contrary to many of the following treatments and seemingly in accordance with the social control theory, the Medical Superintendent exercised absolute control over this treatment, starting with the selection of suitable patients to the length of treatment deemed necessary. Patients as well as their relatives seemed to have accepted the Superintendent's decision without question. The late-1930s witnessed the decline of sleep therapies in Devon, possibly due to other upcoming therapies, but, as the following quotation illustrates, not only were they falsely regarded as ‘treatments’, but also as the only available intervention: ‘sleep cures [were] the only treatment we had’.^74^ In 1941, somnifaine treatment ‘[was still] occasionally used in very excited patients and has given quite good results’.^75^ It remained in use for very excited patients unsuitable for electro-convulsive therapy.

## Fever therapy

In the late 19th and early 20th centuries, patients suffering from the terminal stages of syphilis placed great strain on nursing staff. When mercurial treatments failed, the DCLA in 1924 took up Julius Wagner-Jauregg's attempt to cure the disease by infecting patients with malaria.^76^ The treatment was not without risk, and, echoing with the arguments of social control theorists, its administration was controlled by the Medical Superintendent who selected suitable patients and the patients’ relatives who signed the consent form, thus leaving the patient without a choice about their treatment. Although voluntary patients could from 1931 legally refuse treatment, they were not eligible to consent to it. By today's standards relatives were hardly in a position of providing informed consent either. The wording on the consent forms was both suggestive and reassuring, claiming the patient would receive the ‘latest method of treatment’ and assuming compliance, all the while carefully avoiding information on any risks involved. It is also noteworthy that before the late 1930s the diagnosis was not included on the form – possibly owing to the great stigma syphilis carried. While, similar to prolonged narcosis, the initiative to apply this treatment came exclusively from the Superintendent, it will be shown later in this paper that subsequent treatments were often demanded by relatives who had heard or read about them. Fever treatment in the DCLA was restricted by limitations of space, as patients had to be isolated for the course of the treatment which was not always possible owing to overcrowding. Despite a considerable risk for patients if they discharged themselves before completing the treatment, no significant differences were made between voluntary and certified patients who received this kind of treatment.^77^ Although the Devon case notes attest to the occasional temporary remission and the patient's discharge, this treatment failed to live up to early ambitious hopes, and the advent of antibiotics eventually confined malaria treatment to history. It did, however, strengthen the notion that ‘affecting the body and maybe the brain in some way could be curative’,^78^ an approach nearly all later treatments built on, and it reinforced ideas about the organic origin of mental disorders.

Similar to the early sedatives described above, malaria therapy also allowed doctors to engage in medical research and enhance their professional reputation, as the treatment doubled as a study into anti-malaria agents. The DCLA, for instance, supported the League of Nations with a trial of quinine, atebrin and a new synthetic preparation, phenoquine, to compare efficacy in the prevention of malaria.^79^ The importance of this research on a national scale can be gleaned from the fact that even in the early 1930s only a small number of British mental hospitals offered malaria treatment.^80^ There is, however, no evidence that either relatives or patients specifically consented to – or were even aware of – these trials.

## ‘Shocking’ patients into sanity

**Figure 4:**
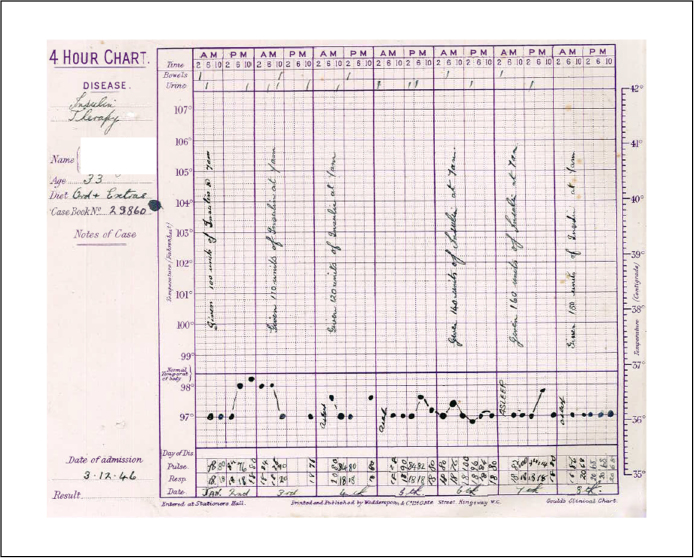
ICT monitor chart.

Malaria therapy was only given to the most severely ill patients and restricted to one form of mental illness, but in the 1930s shock therapies emerged that allowed doctors to treat less severe forms of mental illness. Hypoglycaemic coma or Insulin Coma Therapy (ICT) was pioneered in Devon in June 1937, two years after the Austrian Manfred Joshua Sakel developed it. Patients started with 20 units of insulin, usually at 7 am, which was then incrementally increased ‘until sweating occur[red]’ – in several cases up to 440 units, and some patients endured as many as 51 comas. During the treatment, patients’ vital signs were monitored on ICT charts, and after waking up, patients received sweetened tea and glucose (Figure 4).

In addition to controlling and modifying a patient's behaviour on the wards, ICT appeared to be a large step towards aligning psychiatry with general medicine. Not only was the treatment based on a hormone, an accepted pharmaceutical agent, but in order to conform to UK regulations, the Devon hospitals, similar to general hospitals, had to establish special treatment units for men and women, a process whose completion took two years.^81^ For the patients the ‘cure’ was short-lived. Most were discharged after a course of ICT, but a large number were readmitted with similar symptoms within the following one or two years, and at least one patient died in Devon from irreversible coma while undergoing ICT. A vivid account of the unpleasantness of this treatment can be found in Wilson.^82^ During the war, with insulin and sugar in short supply, ICT treatment in Devon declined, but increased again towards the end of the 1940s and correspondence suggests that there were waiting lists for vacancies in the Insulin Ward.^83^ The Medical Superintendent seemed pleased to report successful treatment to the relatives, but remained cautious about long-term prospects.^84^ The following years confirmed that the Devon doctors had been over-enthusiastic about ICT whose efficacy was scientifically unproven and soon refuted.^85^

Chemical shock therapies, allegedly dating back to Paracelsus’ attempt to treat insanity through seizures induced by oral administration of camphor,^86^ did not enter mainstream psychiatry before 1934. The fact that psychotic epileptics became lucid during fits^87^ suggested that seizures might cure psychoses,^88^ and the Hungarian Ladislas von Meduna introduced shock by Cardiazol, a camphor-like substance. He achieved only modest results, observing no change in as many patients as he reported improved or recovered.^89^ Despite its immediate dangers, including death from ‘status epilepticus’, joint dislocations and hairline fractures in the spine – amplified by the patient's fear of the treatment – in 1941 fourteen Devon patients received such treatment whose ‘value is beyond doubt’^90^ and the success of the treatment was enthusiastically relayed to relatives. Frequently, the ‘recovery’ did not last long and patients were readmitted to receive more injections. Relatives readily agreed to patients being injected with Cardiazol and while this seems disturbing from today's perspective, it must be acknowledged that, as with fever therapy, risks involved in the treatment were grossly understated. Cardiazol therapy is a prime example of medical staff exercising their authority and seemingly exclusive knowledge over lay people, as it was put to relatives that Cardiazol therapy was considered ‘as the patient is not re-acting [sic] satisfactorily to the usual methods of treatment’, suggesting Cardiazol therapy was a last resort if improvement was expected. On many occasions it was not evident to which kind of shock therapy the relative was consenting. A form used in the early 1940s, for example, asked for ‘consent to commence a course of Electrical or Cardiazol therapy’.^91^

Inducing shock by passing electricity through the patient's body – electro-convulsive treatment (ECT) – was subsequently regarded as much safer.^92^ Developed in 1938 by Ugo Cerletti and Lucio Bini, ECT was introduced in Devon in 1943^93^ after a trial period at the Bristol-based Burden Neurological Unit. ECT required minimal staff training and could be administered to a large number of patients in a short time period, even at out-patient appointments. It therefore complied with the demands of reducing costs. Furthermore, it was fully controllable by medical staff,^94^ did not entail the adverse reactions to Cardiazol shock therapy due to instant loss of consciousness, and relaxants minimised the risk of bone fractures. First results in Devon seemed promising, after the electro-convulsive apparatus ‘gave a little trouble […] through faulty connections’.^95^ Several fractures of the spinal vertebrae were recorded during the first two years of usage, but the Medical Superintendent quickly assured that the patients concerned were ‘now up and about in a plaster jacket [and] quite comfortable’.^96^ Patients and relatives might have seen this differently. One woman who had agreed to ECT and was later informed that her husband had fractured ‘three bones in the vertebrae column’ responded that she had feared that this might happen, but did not want to affect his recovery by withholding consent. ECT did not have the same effect in all patients, and the Medical Superintendent was reluctant to make statements regarding a patient's future prospects.^97^ In spite of this, ECT is the only shock treatment whose efficacy has been scientifically established^98^ and that survived until the present day, although for a much smaller number of patients who fail to respond to other therapies.

**Figure 5:**
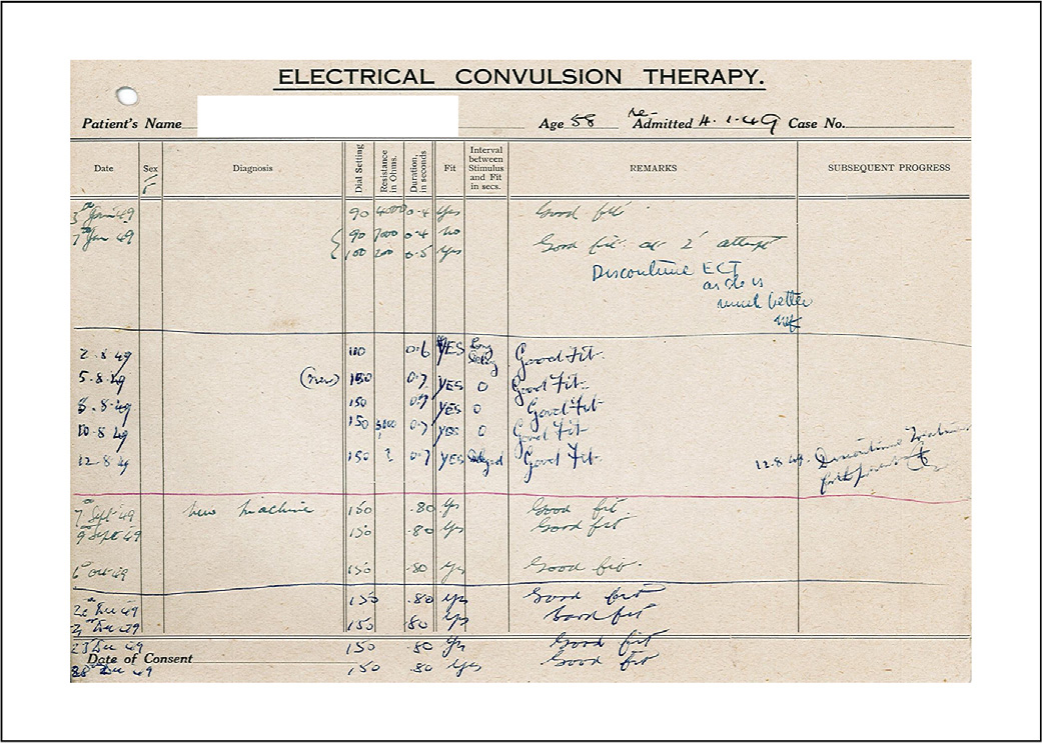
ECT treatment card.

Although originally introduced for people suffering from schizophrenia, the use of ECT in Devon swiftly shifted towards diagnoses of depression. By 1944 it had become clear that ‘the best results [are] obtained in the depressed and confused types’, and that despite some patients who did not respond to the treatment ‘the results on the whole [are] very gratifying’.^99^ ECT was widely used in Devon, as in all other British mental hospitals, during the 1940s and early 1950s, primarily in female patients, as its (temporary) success reduced the duration of hospitalisation significantly and a calming effect on patients could be observed. Doctors were particularly pleased to report that female patients treated with ECT regained the ability to fulfil their social function as housewives, managing their household satisfactorily again. Treatments were usually given in two to three day intervals and recorded on special charts (Figure 5).

Its use declined with the advent of drug treatment, but interestingly, the late 1960s witnessed a resurgence of ECT – possibly because the limitations of the drugs had been recognised by then and more detailed research allowed for better evaluation of its efficacy. Many a patient went through several courses of ECT during their stay in the Devon hospitals. Shock therapies seem to have been less agreeable with patients than staff. As with fever therapy and ICT, consent for the treatment was sought from the next-of-kin, but case notes dating from the late 1940s and early 1950s give evidence that several voluntary patients either refused ECT altogether or accepted a few treatments, but refused further. It is remarkable how many relatives agreed to ECT admitting utter ignorance of its nature, trusting the Medical Superintendent would act in the patient's best interest. Others consented to ECT knowing the patient would be hesitant or had already refused it, but here again trust in the Medical Superintendent and hope for a cure seem to have outweighed any concerns. This clearly refutes the idea of mental hospitals as ‘dumping grounds’ for unwanted relatives as outlined by some social control theorists. ECT and psychosurgery – described in the following paragraph – were the treatments most frequently requested by relatives for their mentally ill family member. This illustrates that while families have always been interested in the return of their relative, they were becoming more knowledgeable about treatments, demanding more active involvement in the patient's therapeutic regime. The surviving archival materials relating to the 1930s and 1940s therefore suggest that the walls of the mental hospital were much more porous than previously assumed. Not only would relatives visit the patients regularly and keep in touch through correspondence, but on many occasions relatives used the visiting day to make an appointment with the Superintendent in order to discuss the patient's treatment – frequently after discussing the same issue with the patient during the visiting time. This again attests to the diminishing control of the Superintendent over treatments administered to his patients and also illustrates the family's efforts to ensure the patient's complete recovery and return home to the community.

## Severing brain connections

The 1930s saw a resurgence of 19th century localised brain surgery based on the assumption that certain brain regions were the sites of mental illness.^100^ In 1935 the Portuguese neurologist Egas Moniz conducted the first ‘leucotomy’ by severing connections between the frontal lobes and the rest of the brain, hoping to alter a patient's behaviour.^101^ This procedure was refined to a ‘lobotomy’ in 1937, thus reducing the risks of psychosurgery. Both methods were eventually discredited^102^ owing to high mortality rates, risks of haemorrhage, and their potential irreversible damage to the patient's personality. This was undoubtedly the most invasive form of controlling a person's behaviour, as ‘every patient probably loses something by this operation, some spontaneity, some sparkle, some flavour of the personality’.^103^ Despite its problems, brain surgery was enthusiastically welcomed in Britain. Between 1942 and 1954 over 10,000 patients underwent the operation in England and Wales,^104^ and more than 40% either recovered or were greatly improved. With hindsight, this figure has to be questioned, as the study was published in 1961, thus did not allow for an assessment of any long-term effects.

Psychosurgery was first trialled in Devon in 1943 with desired benefit for both patients and staff: ‘Certain types of mental patients react well such as chronic melancholics and patients who have become degraded in habits. To effect an improvement in these types of patients also relieves the nursing staff of very unpleasant duties’. The Medical Superintendent was intent upon the continuation of these operations, admitting that they were merely at an experimental stage: ‘I am now making a selection of patients of both sexes who are chronically ill and who are very difficult to nurse, and propose to have the operation done, if the consent of the relatives is given. After that series is completed it will be possible to assess the results. This is a very new and experimental form of treatment and is not without danger but the risk is more than worthwhile in the type of patients selected. […] I think it would be fair if I asked the relatives to contribute towards the cost of the operation.’^105^ Although he recognised the need for critical assessment, his study will have been compromised by the selection of a minority of patients who, despite several other therapies, remained extremely disturbed as well as the lack of evidence on potential long-term consequences. Given the surgical risks involved and the experimental stage of the treatment it seems preposterous that relatives were asked to support the operation financially. Several more female patients were treated in the following months, and the first male patient in August 1944. But the Superintendent had to admit in the same year that ‘leucotomy has been performed in five patients up to date, but so far without any striking success’.^106^ Only a minority of Devon patients left the hospital recovered after the operation, several died, a few were relieved of their symptoms and several re-entered the hospital with ‘post-leucotomy personality disorder’. While the annual reports suggest that the Superintendent had absolute power over which patient was selected for the operation, it must be noted that correspondence increasingly contained evidence that psychosurgery was requested by relatives, particularly after the award of the Nobel Prize to Moniz had been broadcast on the radio. The reason for the comparatively few operations carried out in the hospitals under consideration was not only the Superintendent's refusal to be pressurised into such operations,^107^ but also the lack of facilities, notably an in-house surgeon at that time. In some cases relatives responded to this by removing their family member to a different institution where the operation could be carried out, thus undermining the Superintendent's authority.

All the above treatments have in common that they were symptomatic, but primarily unspecific attempts to ameliorate mental disturbances and that patients had very little control about which treatment they received. The discovery of the first modern psychotropic drugs did not alter this. Since the 1950s, drugs with severe side effects have been developed and, contrary to their forbearers, continue to be prescribed today. Since the advent of the first psychotropic drugs very few new drugs have been introduced – any ‘new’ medication was usually based on the modification of an older substance with a view towards reducing side effects and other risks. Nevertheless, advocates went as far as identifying the advent of drug treatment uncritically as the third revolution in psychiatry.^108^ It is undeniable, however, that the advent of psychotropic drugs reduced the costs of treating mental disorders and facilitated the medicalisation of mental illness.

## The ‘magic bullet’

The calming effect of Largactil,^109^ the first anti-psychotic, was discovered serendipitously in 1950 by French scientists searching for an anti-histamine to treat cardio-respiratory shock. It was later found that Largactil has severe adverse effects on motor functions, and patients required a ‘cocktail’ of various drugs to relieve the side effects. Nevertheless, starting in 1954, Largactil was tried on almost all patients in Devon, irrespective of their diagnoses, thus underlining the drug's non-specificity. Case notes reveal that in some instances Largactil could be used to facilitate patient management. As with the early sedatives, patients did not have to take psychotropic drugs as long as they were ‘quiet and co-operative’ – any instance of disruptive or noisy behaviour, however, was swiftly brought under control through a course of Largactil or later its successors. Doses administered to severely disturbed patients could exceed the recommended daily dose of 150–200 mg^110^ by three or four times.

The development of Largactil coincided with the rediscovery of *Rauwolfia serpentina* whose active ingredient, *reserpine*, had a calming effect on psychotic patients. Available in Devon from 1955, this drug's use declined significantly towards the late 1950s when other psychotropic drugs, including antidepressants and anxiolytics (anti-anxiety drugs), were developed in quick succession. Although mood stabilisers such as *lithium carbonate* had been introduced to psychiatry in 1949,^111^ they lived a shadow life in Devon up to the 1960s, and even then their use remained minimal compared to the use of other psychotropic drugs. By 1960, Devon used 11 antipsychotics, 6 antidepressants, and 5 anxiolytics. Amongst the latter, *meprobamate* introduced in 1956 was the first one on the market. It was followed by Librium which had been synthesised from *chlorpromazine* in 1955,^112^ Valium and Ativan. With the only alternatives being the more toxic barbiturates or early neuroleptics with their severe side effects, anxiolytics were regarded as extremely safe when first introduced. They have since been referred to ‘as one of the greatest menaces to society in peacetime, as coming off them is harder than coming off heroin’.^113^ Systematic research into structurally similar drugs to *chlorpromazine* yielded more potent drugs, requiring smaller doses and providing longer action, thus freeing nursing staff for other duties. By 1970, the number of antipsychotics used in Devon had increased to 27, that of antidepressants to 29, and that of anxiolytics only slightly to 8. Despite more potent drugs being on the market, Largactil continued to be used widely. Contrary to antipsychotics and anxiolytics, antidepressants established themselves only gradually on the medical market, constantly competing with ECT.^114^

The large number of drugs available from the mid-1960s allowed frequent medication changes – often in very short intervals, reminiscent of the various unproven treatments administered during the first half of the twentieth century, leaving the impression that doctors were still experimenting. Although psychopharmacotherapy seemingly benefited patients as well as nursing staff through enhancing patients’ social capabilities, reducing expensive, long-term hospitalisation, and avoiding damage through invasive surgery, some patients showed minimal or no improvement at all. Drugs also allowed patients to take more control over their therapeutic regime. It was observed that while in hospital, ‘they hide it under their pillows, spit it out when unobserved, beg to have it discontinued. They argue it makes them feel sleepy, feel sick, gain weight […]’.^115^ The Devon case files provide ample evidence of patients’ expressing their concerns about drugs and sometimes their refusal to take them. In many cases staff responded by changing medication from tablets to injections. It is interesting to note that, contrary to the shock treatments and psychosurgery, no specific consent from either patients or relatives was sought before drug therapy was started, usually immediately after admission.

Once discharged into the community it was even easier for patients to tamper with their medication, which, depending on the severity of the illness and the degree of dependency, often required rehospitalisation. At the same time, society, frightened through media coverage of incidents caused by discharged patients, expected greater conformity with social norms, thus demanding tighter measures to control the mentally ill in the community. It might have been partly as a response to this that ‘depot medication’, such as Moditen and Modecate^116^ was introduced from 1968 in Devon. Contrary to tablets or syrups, these injections release the drug slowly, thus acting for up to a month. The benefits for the patients included not having to remember taking oral medication on a daily basis, and if they missed an appointment for an injection, they received a reminder. However, some patients felt that they were being subjected to an outside control over their body, ensuring their compliance with a prescribed treatment plan, a feeling that had not been expressed explicitly relating to any of the earlier treatments – refusal of treatment was usually motivated by its unpleasant side effects. The introduction of compulsory treatment orders under the 1983 Mental Health Act has increased this feeling of control imposed by society and medical and legal authorities.

The variety of (unproven) treatments offered to patients in Devon confirms that research into psychiatric treatments intensified during the first half of the 20th century. Fennell referred to the years between 1930 and 1959 as the ‘age of experimentation’,^117^ where treatments would be liberally tried, a fact clearly corroborated by the Devon case notes. At the time of their discharge, many patients had undergone a variety of somatic treatments. Entries on medicine cards specifying ‘trial drugs’ suggest that the Devon mental hospitals continued to participate in trials during the latter half of the 20th century, though no corroborating evidence can be found in the case notes.

## The road to deinstitutionalisation in Devon

By the 1950s and 1960s, when social control theorists began to publish their analyses of 19th century institutions, long-term hospitalisation attracted increasing criticism for its repressiveness and detrimental effects on patients,^118^ stimulating a national move away from the large mental hospitals. Psychiatric out-patient services, introduced under the 1930 MTA and greatly stimulated with the foundation of the NHS, coupled with psychoactive drugs, in particular depot medication,^119^ enabled an ‘open-door’ policy, and Bucknill's vision of ‘boarding-out’ was finally put into practice with day hospitals and hostel-based treatment. The role of drugs in this process, however, is frequently overrated, ignoring their negative effects on patients and staff.^120^ The introduction of ECT had already enabled out-patient treatment, and in Devon in-patient treatment had shortened considerably with the introduction of voluntary treatment under the 1930 MTA and more vigorously after the foundation of the NHS. This confirms that politics played a major role in the final closure of British mental hospitals.

According to Health Minister Enoch Powell's 1962 *National Hospital Plan for England and Wales*, based solely on the results and statistical predictions on one study,^121^ the DCLA, then known as Exe Vale Hospital and combined with Digby and Wonford House, was expected to almost halve the number of beds by 1975. The hospital eventually fell victim to closure in 1986. While the former buildings of the DCLA and Digby have been converted into apartments, Wonford House has since been acting as headquarter of the Devon NHS Trust. Devon currently has 24 beds available for the treatment of acutely disturbed patients. Provisions for the chronically mentally ill are much bleaker with long waiting lists, placing again the burden of care into the hands of relatives and limiting individual treatment options. A report commissioned by the Devon Primary Care Trust found that, although ‘internationally renowned for its deinstitutionalisation in the 1980s, efforts to continue the process seem to have stalled [in Devon]’.^122^ The report, which also takes into account service users criticises that ‘home-based treatment […] sometimes amounted to just a daily phone call or a visit to give people their medication’, a fact the Trust is on course to have remedied by 2015.

## Conclusion

The 20th century witnessed major changes in the legislative and therapeutic framework applicable to people suffering from mental disorders, aiming to reduce outside control and grant patients greater agency in their treatment. The DCLA and the treatments provided therein are in many ways representative of these developments. The hospital was erected when mental illness was thought to be curable in segregated institutions through a regime of strict discipline, routine and supervision. Families had been involved in the processes of committal and discharge since the foundation of the DCLA, but their influence increased in the 20th century when the therapeutic regime became part of the negotiations between the institution and lay people. Social control has never vanished entirely from mental health care, but contrary to the social control theorists who saw the power with the medical profession, it shifted increasingly to third parties. These developments were accompanied by an increasing medicalisation of mental illness, allowing doctors to trial myriad treatments for whose efficacy existed very limited evidence,^123^ with the consent of patients’ relatives. Apart from the lack of a convincing explanation as to how these treatments actually worked, there were other commonalities. The main goal of their application was to alter patient behaviour. In fact, the Devon case notes reveal that the success of a treatment was often measured in a patient's ability to dress and feed themselves adequately, take part in social activities and daily work, i.e. the patient's conformity with accepted social norms and roles. Rather than being the passive victims of experimentation, as Scull provocatively – and prematurely – suggested, patients and their relatives began to expect, sometimes demand the new, often hazardous, treatments. Expectations were frequently fuelled by enthusiastic reports compiled by contemporaries as well as upcoming media influence which guided public opinion and community demand. At the same time legislative changes demanded that doctors carry out research in frequently ill-equipped institutions. Careful analysis of surviving documents illustrates that such pressures did not lead to an orgy of experimentation in Devon, as patients were carefully selected for the procedures – apart from drug treatment which did not require specific consent. It also reveals that the therapeutic regime was often compromised by institutional, legislative and social factors, preventing patients from acting as full consumers. Contrary to the aims of governmental reform, deinstitutionalisation did not necessarily decrease outside control over the mentally ill, but shifted the burden of care onto families, the closure of the large mental hospitals leaving fewer treatment options for patients.

## Footnotes

2 Case paper 15775, Devon Record Office, Exeter.

3 From the mid-19th century Devon had three mental hospitals, all located in or near the City of Exeter. The Devon County Lunatic Asylum (DCLA, later renamed ‘Exminster Hospital’) accepted patients from all over the county. Exeter City Asylum (later ‘Digbys’) catered for the population of the City of Exeter, and Wonford House for private patients. This article builds mainly on materials from the DCLA, but includes aspects of the other two institutions, as in 1963 all three hospitals merged into Exe Vale Hospital, which finally closed its doors in 1986.

4 Forsythe B, Melling J, Adair R. The new poor law and the county pauper lunatic asylum – the Devon experience 1834–1884. *The Social History of Medicine* 1996;**9**:335–355.

5 Castel R. Moral treatment: Mental therapy and social control in the nineteenth century. In Cohen S, Scull A, editors. *Social control and the state: historical and comparative essays*. London: Blackwell; 1986, p. 248–266.

6 Conrad P, Schneider J. *Deviance and medicalization: from badness to sickness*. Philadelphia: Temple University Press; 1992.

7 Scull A. *The most solitary of afflictions: Madness and Society in Britain, 1700–1900*. New Haven: Yale University Press; 1993; Scull A. *Museums of madness: the social organization of insanity in nineteenth-century England*. London: Allen Lane; 1979.

8 Grob G. *The mad among us. A history of the care of America's mentally ill*. New York: The Free Press; 1994.

9 Shorter E. A *History of Psychiatry: from the era of the asylum to the age of Prozac*. New York: John Wiley & Sons; 1997.

10 Grob G. *The mad among us. A history of the care of America's mentally ill*. New York: The Free Press; 1994.

11 Foucault M. *Madness and Civilisation. A history of insanity in the age of reason*. London: Tavistock Publications; 1971.

12 Goffman E. *Asylums: Essays on the social situation of mental patients and other inmates*. Harmondsworth: Penguin; 1968.

13 Rothman D. *The Discovery of the Asylum. Social order and disorder in the new Republic*. Aldine Transaction: New Brunswick & London; 2001.

14 Rothman D. *The Discovery of the Asylum. Social order and disorder in the new Republic*. Aldine Transaction: New Brunswick & London; 2001; Scull A. *Museums of madness: the social organization of insanity in nineteenth-century England*. London: Allen Lane; 1979.

15 Conrad P. The shifting engines of medicalization. *Journal of Health and Social Behaviour* 2005;**46**:3–14.

16 Scull A. *Museums of madness: the social organization of insanity in nineteenth-century England*. London: Allen Lane; 1979; Scull A. Humanitarianism or Control? Some Observations on the Historiography of Anglo-American Psychiatry. In Cohen S, Scull A, editors. *Social control and the state: historical and comparative essays*. London: Blackwell; 1986, p. 118–140.

17 Shorter E. A *History of Psychiatry: from the era of the asylum to the age of Prozac*. New York: John Wiley & Sons; 1997.

18 Scull A. *Museums of madness: the social organization of insanity in nineteenth-century England*. London: Allen Lane; 1979.

19 Scull A. *The insanity of place/the place of insanity: essays on the history of psychiatry*. London: Routledge; 2006.

20 Cherry S. *Mental Health Care in Modern England: the Norfolk Lunatic Asylum/St. Andrew's Hospital c.1810-1998*. Suffolk: Boydell Press; 2003; Scull A. A convenient place to get rid of inconvenient people: TheVictorian Lunatic Asylum. In King A, editor. *Buildings and Society*. London: Routledge & Kegan Paul; 1980, p 37–60; Digby A. Changes in the asylum: The case of York 1777–1815, *The Economic History Review* 1983;**36**:218–39; Digby A. *Madness, morality, and medicine: a study of the York Retreat, 1796–1914*. Cambridge: CUP; 1985; MacKenzie C. *Psychiatry for the Rich. A History of Ticehurst Private Asylum, 1792–1917*. London: Routledge; 1992; Michaels P, Hirst D. Establishing the ‘rule of kindness’: the foundation of the North Wales Lunatic Asylum, Denbigh. In Melling J, Forsythe B, editors. *Insanity, institutions and society, 1800–1914*. London: Routledge; 1999, p. 159–179; Gittins D. *Madness in its place: narratives of Serveralls Hospital, 1913–1997*. London: Routledge; 1998; Pearce D. Family, gender and class in psychiatric patient care during the 1930s: The 1930 Mental Treatment Act and the Devon Mental Hospital. In Dale, P, Melling J, editors. *Mental illness and learning disability since 1850*. London: Routledge; 2006, p. 112–130.

21 Doerner K. Madmen and the Bourgeoisie: *A Social History of Insanity and Psychiatry*. Oxford: Basil Blackwell; 1981 (translated by Joachim Neugroschel and Jean Steinberg); Prestwich P. Family strategies and medical power: ‘voluntary’ committal in a Parisian asylum, 1876–1914. *Journal of Social History* 1994;**27**:799–818; Finnane M. *Insanity and the Insane in Post-Famine Ireland*. London: Croom Helm; 1981.

22 Tomes N. *A generous confidence: Thomas Story Kirkbridge and the art of asylum keeping, 1840–1883*. London & New York: CUP; 1984; Dwyer E. *Homes for the mad: life inside two nineteenth-century asylums*. New Brunswick: Rutgers University Press; 1987.

23 Adair R, Melling J, Forsythe B. Migration, family structure and pauper lunacy in Victorian England: Admissions to the Devon County Pauper Lunatic Asylum, 1845–1900. *Continuity and Change* 1997;12:373–401.

24 Walton J. Casting out and bringing back in Victorian England: Pauper lunatics 1840–70. In Bynum W, Porter R, Shepherd M, editors. *The Anatomy of Madness*. Volume II. London: Tavistock; 1985, p. 137–141; Pearce D. Family, gender and class in psychiatric patient care during the 1930s: The 1930 Mental Treatment Act and the Devon Mental Hospital. In Dale, P, Melling J, editors. *Mental illness and learning disability since 1850*. London: Routledge; 2006, p. 112–130; Goffman E. The moral career of the mental patient, *Psychiatry* XXII (May 1959), 123–42; Sampson H, Messinger S, Towne A. Family processes and becoming a mental patient. *American Journal of Sociology* 1962;**68**:88–96.

25 Tomes N. *A generous confidence: Thomas Story Kirkbridge and the art of asylum keeping, 1840–1883*. London & New York: CUP; 1984; Walton J. Lunacy in the Industrial Revolution: a study of asylum admissions in Lancashire 1848–1850. *Journal of Social History* 1979;**13**:1–22; Wright D. Familial care of ‘idiot’ children in Victorian England. In Horden, P, Smith R, editors. *The locus of care. Families, communities, institutions, and the provision of welfare since antiquity*. London: Routledge; 1997, p. 176–197.

26 Sampson H, Messinger S, Towne A. Family processes and becoming a mental patient. *American Journal of Sociology* 1962;**68**:88–96.

27 Tomes N. The Anglo-American Asylum in Historical Perspective. In Smith C, Giggs J, editors. *Location and Stigma: Contemporary Perspectives on Mental Health and Mental Health Care*. Boston: Unwin Hyman; 1988, p. 3–20.

28 Finnane M. Asylums, families and the state. *History Workshop* 1985;**20**:134–148.

29 Adair R, Melling J, Forsythe B. Migration, family structure and pauper lunacy in Victorian England: Admissions to the Devon County Pauper Lunatic Asylum, 1845–1900. *Continuity and Change* 1997;12:373–401; Prestwich P. Family strategies and medical power: ‘voluntary’ committal in a Parisian asylum, 1876–1914. *Journal of Social History* 1994;**27**:799–818; Murphy E. Workhouse care of the insane, 1845–1890. In: Dale P, Melling J. *Mental illness and learning disability since 1850*. London: Routledge; 2006; Wright D. Getting out of the asylum: understanding the confinement of the insane in the nineteenth century. *Social History of Medicine* 1997;**10**:137–155; Wright D. The discharge of pauper lunatics from county asylums in mid-Victorian England: the case of Buckinghamshire, 1853–1872. In Melling J, Forsythe B, editors. *Insanity, institutions and society, 1800–1914: A social history of madness in comparative perspective*. London: Routledge; 1999, p. 93–112; Melling R, Adair, R, Forsythe, B. A proper lunatic for two years: pauper lunatic children in Victorian and Edwardian England: child admissions to the Devon County Lunatic Asylum, 1845–1914. *Journal of Social History* 1997;**31**:371–405.

30 Suzuki A. Enclosing and disclosing lunatics within the family walls: domestic psychiatric regime and the public sphere in early nineteenth-century England. In Bartlett P, Wright D, editors. *Outside the walls of the asylum. The history of care in the community 1750–2000*. London: Athlone; 1999, p. 115–131; Melling J, Forsythe B, Adair R. Families, communities and the legal regulation of lunacy in Victorian England: assessments of crime, violence and welfare in admissions to the Devon Asylum, 1845–1914. In Bartlett P, Wright D, editors. *Outside the walls of the asylum. The history of care in the community 1750–2000*. London: Athlone; 1999, p. 153–180; Melling J, Forsythe B. *The politics of madness. The state, insanity and society in England, 1845–1914*. London: Routledge; 2006.

31 Robertson L. The alleged increase of lunacy. *Journal of Mental Science* 1869;**15**:1–23; Maudsley H. Is insanity on the increase? *British Medical Journal* 1871;**1**:36–39; Maudsley H. The alleged increase of insanity. *Journal of Mental Science* 1877;**23**:45–54; Humphreys N. Statistics of Insanity in England, with special reference to its alleged increasing prevalence. *Journal of the Royal Statistical Society* 1890;**53**:201–252; Jarvis E. On the supposed increase in insanity. *American Journal of Insanity* 1852;**8**:333–364; Hare, E. Was insanity on the increase? The fifty-sixth Maudsley Lecture. *British Journal of Psychiatry* 1983;**142**:439–55.

32 Medical Superintendent's Annual Reports, Devon Record Office, 1872.

33 Scull A. *The most solitary of afflictions: Madness and Society in Britain, 1700–1900*. New Haven: Yale University Press; 1993; Foucault M. *Madness and Civilisation. A history of insanity in the age of reason*. London: Tavistock Publications; 1971; Goffman E. *Asylums: Essays on the social situation of mental patients and other inmates*. Harmondsworth: Penguin; 1968; Doerner K. Madmen and the Bourgeoisie: *A Social History of Insanity and Psychiatry*. Oxford: Basil Blackwell; 1981 (translated by Joachim Neugroschel and Jean Steinberg).

34 Scull A. *The most solitary of afflictions: Madness and Society in Britain, 1700–1900*. New Haven: Yale University Press; 1993.

35 MacDonald M. Madness, suicide and the computer. In Porter R, Wear A, editors. *Problems and methods in the history of medicine*. New York: Croon Helm; 1987, p. 207–229; Grob G. *The mad among us. A history of the care of America's mentally ill*. New York: The Free Press; 1994.

36 5th Annual Report of Inspectors of Lunatics, 1851, p. 6.

37 Medical Superintendent's Annual Reports, Devon Record Office, 1854, p. 11.

38 Mortimer Granville J. *The care and cure of the insane*. London: Hardwicke & Bogue; 1877.

39 Correspondence regarding GH, DRO.

40 Correspondence regarding GR, DRO.

41 Medical Superintendent's Annual Reports, Devon Record Office, 1944.

42 Pearce D. *The operation of the 1930 Mental Treatment Act in local psychiatric hospitals. The introduction of voluntary patients and new treatment regimes in the Devon Mental Hospital, 1931–1938*. Unpublished PhD thesis, 2002.

43 MacKenzie C. Social factors in the admission, discharge and continuing stay of patients at Ticehurst Asylum, 1845–1917. In Bynum W, Porter R, Shepherd M, editors. *The Anatomy of Madness*. Volume II. London: Tavistock; 1985, p. 147–174.

44 Wright D. Getting out of the asylum: understanding the confinement of the insane in the nineteenth century. *Social History of Medicine* 1997;**10**:137–155.

45 Curtis R. Some developments in mental treatment. *The Journal of Mental Science* 1938:183–202.

46 Melling J, Forsythe B. *The politics of madness. The state, insanity and society in England, 1845–1914*. London: Routledge; 2006.

47 Medical Superintendent's Annual Reports, Devon Record Office, 1848 & 1850.

48 Foucault M. *Madness and Civilisation. A history of insanity in the age of reason*. London: Tavistock Publications; 1971, p. 278.

49 Goffman E. *Asylums: Essays on the social situation of mental patients and other inmates*. Harmondsworth: Penguin; 1968.

50 Rothman D. *The Discovery of the Asylum. Social order and disorder in the new Republic*. Aldine Transaction: New Brunswick & London; 2001.

51 Bucknill J, Tuke DH. A manual of psychological medicine. Publisher unknown; 1879.

52 The Annual Report of the Committee 1848–50.

53 Digby A. *Madness, morality, and medicine: a study of the York Retreat, 1796–1914*. Cambridge: CUP; 1985, p.76.

54 Shepherd A, Wright D. Madness, Suicide and the Victorian Asylum: Attempted Self-Murder in the Age of Non-Restraint. *Medical History*, 2002;**46**:175–196.

55 Interviews with Miss o’Brien and Dr Jack, Devon Record Office.

56 Correspondence regarding FN, Devon Record Office.

57 Finnane M. *Insanity and the Insane in Post-Famine Ireland*. London: Croom Helm; 1981.

58 Fennell P. *Treatment without Consent: Law, psychiatry and the treatment of mentally disordered people since 1845*. London: Routledge; 1996; Scull A. *The most solitary of afflictions: Madness and Society in Britain, 1700–1900*. New Haven: Yale University Press; 1993.

59 Grob G. *The mad among us. A history of the care of America's mentally ill*. New York: The Free Press; 1994, p. 85.

60 Cameron R. The philosophy of restraint in the management and treatment of the insane. *Journal of Mental Science* 1881–2;**28**:519.

61 Jones K. *Mental Health and Social Policy 1845–1959*. London: Routledge; 1960.

62 Allan, S *Devon Mental Hospital 1845–1945. The History of the Hospital*. Publisher unknown; 1945, p. 39.

63 Grob G. *The mad among us. A history of the care of America's mentally ill*. New York: The Free Press; 1994.

64 Shorter E. A *History of Psychiatry: from the era of the asylum to the age of Prozac*. New York: John Wiley & Sons; 1997.

65 Valenstein E. *Great and desperate cures: the rise and decline of psychosurgery and other radical treatments for mental illness*. New York: Basic Books; 1986; Scull A. Somatic treatments and the historiography of psychiatry. *History of Psychiatry* 1994;**5**:1–12.

66 Braslow J. Punishment or therapy: patients, doctors and somatic remedies in the early twentieth century. *Psychiatric Clinics of North America* 1994;**17**:493–513; MacKenzie C. *Psychiatry for the rich: a history of Ticehurst Private Asylum, 1792–1914*. London: Routledge; 1993.

67 Correspondence regarding FS, Devon Record Office.

68 Eager, R. *Hints to probationer nurses in mental hospitals*. 3rd edition. London: HK Lewis; 1939, p. 210.

69 Valenstein E. *Great and desperate cures: the rise and decline of psychosurgery and other radical treatments for mental illness*. New York: Basic Books; 1986.

70 Dear M, Taylor S. *Not on our Street: community attitudes to mental health care*. London: Pion; 1982, p. 157.

71 Dear M, Taylor S. *Not on our Street: community attitudes to mental health care*. London: Pion; 1982.

72 Pinfold V. Is compulsory community treatment ever justified?. *The Psychiatrist* 2001;**25**:268–270; Lawton-Smith S. Community treatment orders are not a good thing. *The British Journal of Psychiatry* 2008;**193**:96–100.

73 Windholz G, Witherspoon L. Sleep as a cure for schizophrenia: an historical episode. *History of Psychiatry* 1993;**4**:83–93.

74 Slater E. Psychiatry in the thirties. *Contemporary Review* 1975;**226**:70–5.

75 Medical Superintendent's Annual Reports, Devon Record Office, 1941.

76 Whitrow M. Wagner-Jauregg and fever therapy. *Medical History* 1990;**34**: 294–310.

77 Curtis R. Some developments in mental treatment. *The Journal of Mental Science* 1938:183–202.

78 Healy D. *The Creation of Psychopharmacology*. London: Harvard University Press; 2002, p. 51.

79 James S. Antimalarial chemotherapeutic tests at the Devon Mental Hospital. *Journal of Tropical Medicine & Hygiene* 1933;**36**:289–91.

80 Pearce D. *The operation of the 1930 Mental Treatment Act in local psychiatric hospitals. The introduction of voluntary patients and new treatment regimes in the Devon Mental Hospital, 1931–1938*. Unpublished PhD thesis, 2002.

81 Medical Superintendent's Annual Reports, Devon Record Office, 1941.

82 Wilson I. A study of hypoglycaemic shock treatment in schizophrenia. London: HMSO; 1936.

83 Correspondence regarding HG, Devon Record Office.

84 Correspondence regarding HG, Devon Record Office.

85 Bourne H. The insulin myth. *Lancet* 1953;**265**: 964–968; Ackner B, Harris A, Oldham A. Insulin treatment of schizophrenia; a controlled study. *Lancet* 1957;**272**: 607–611.

86 Dubovsky S. Electroconvulsive therapy. In: Kaplan H, Sadock J, editors. *Comprehensive Textbook of Psychiatry*. Baltimore: Williams and Wilkins; 1995, p 2129–40.

87 Kennedy A. Convulsion therapy in schizophrenia. *Journal of Mental Science* 1937;**83**: 609–629.

88 McCrae N. ‘A violent thunderstorm’: Cardiazol treatment in British mental hospitals. *History of Psychiatry* 2006;**17**: 67–90.

89 Valenstein E. *Great and desperate cures: the rise and decline of psychosurgery and other radical treatments for mental illness*. New York: Basic Books; 1986.

90 Medical Superintendent's Annual Reports, Devon Record Office, 1941.

91 Correspondence regarding EB, Devon Record Office.

92 Fleming G, Golla F, Walter W. Electric-convulsion therapy of schizophrenia. *Lancet* II 1939, 1353–5.

93 Interview with Dr Jack, Devon Record Office.

94 Fleming G, Golla F, Walter W. Electric-convulsion therapy of schizophrenia. *Lancet* II 1939, 1353–5; Shepley W, McGregor, J. The clinical applications of electrically induced convulsions. *Proceedings of the Royal Society of Medicine* 1940;**30**:267–9.

95 Medical Superintendent's Monthly Reports, Devon Record Office, April 1943.

96 Medical Superintendent's Monthly Reports, Devon Record Office, March & April 1944.

97 Correspondence regarding EW, Devon Record Office.

98 Janicak P, Davis J, Preskorn S, Ayd F. *Principles and Practice of Psychopharmacotherapy*, 2nd ed. Baltimore: Williams & Wilkins; 1997.

99 Medical Superintendent's Annual Reports, Devon Record Office, 1944, p. 17.

100 Joanette Y, Stemmer B, Assal G et al. From Theory to Practice: the unconventional contribution of Gottlieb Burckhardt to psychosurgery. *Brain Language* 1993;**45**:572–87; Shorter E. A *History of Psychiatry: from the era of the asylum to the age of Prozac*. New York: John Wiley & Sons; 1997; Berrios G. Psychosurgery in Britain and elsewhere: a conceptual history. In: Berrios G, Freeman H, editors. *150 Years of British psychiatry, 1841–1991*. London: Gaskell; 1991, p. 180–196.

101 Berrios G. Psychosurgery in Britain and elsewhere: a conceptual history. In: Berrios G, Freeman H, editors. *150 Years of British psychiatry, 1841–1991*. London: Gaskell; 1991, p. 180–196.

102 Valenstein E. *Great and desperate cures: the rise and decline of psychosurgery and other radical treatments for mental illness*. New York: Basic Books; 1986.

103 Freeman W, Watts J. Prefrontal lobotomy in the treatment of mental disorders. *Southern Medicine Journal* 1937;**30**: 23–31.

104 Tooth G, Newton M. *Leukotomy in England and Wales 1942–1954*. London, Her Majesty's Stationary Office, 1961.

105 Medical Superintendent's Monthly Reports, Devon Record Office, April 1943.

106 Medical Superintendent's Annual Reports, Devon Record Office, 1944, p. 17.

107 Correspondence regarding ES, Devon Record Office.

108 Swazey J. *Chlorpromazine in Psychiatry: a study of therapeutic innovation*. Cambridge: MIT Press; 1974.

109 UK tradenames are used in this article when referring to medication.

110 Baker A. Observations on the effect of Largactil in psychiatric illness. *Journal of Mental Science* 1955;**101**:175–182.

111 Mitchel P, Hadzi-Pavlovic D, Manji H. Fifty years of treatment for bipolar disorder: a celebration of John Cade's discovery (editorial comment). *Australia & New Zealand Journal of Psychiatry* 1999;**33**:S1–122.

112 Rogers A, Pilgrim D, Brennan S, Sulaiman I, Watson G, Chew-Graham C. Prescribing benzodiazepines in general practice: a new view of an old problem. *Health* 2007;**11**:181–198.

113 Healy D. *Psychiatric drugs explained*. London: Churchill Livingstone; 2003.

114 Healy D. *Psychiatric drugs explained*. London: Churchill Livingstone; 2003.

115 Slear G. Psychiatric patients: clinically improved, but socially disabled. *Social Work* 1959;**4**:66.

116 Cookson J, Taylor D, Catona C. *Use of drugs in psychiatry: the evidence from psychopharmacology*. 5th Revised edition. Cromwell Press Ltd: Trowbridge; 2002.

117 Fennell P. *Treatment without Consent: Law, psychiatry and the treatment of mentally disordered people since 1845*. London: Routledge; 1996.

118 Goffman E. *Asylums: Essays on the social situation of mental patients and other inmates*. Harmondsworth: Penguin; 1968; Houston F. A project for a mental-health village settlement. *Lancet* 1955;**266**:1133–4.

119 Shorter E. A *History of Psychiatry: from the era of the asylum to the age of Prozac*. New York: John Wiley & Sons; 1997; Scull A. *Social order/mental disorder: Anglo-American Psychiatry in historical perspective.* Berkeley: University of California Press; 1989.

120 Szasz T. Some observations on the use of tranquilizing drugs. *AMA Archives of Neurology and Psychiatry*. 1957;**77**:91.

121 Tooth G, Brooke E. Trends in the mental hospital population and their effect on future planning. *Lancet* 1961, 710–713.

122 O’Hagan, M. Adult mental health services in Devon: a high level recovery assessment. 2008. Report commissioned by the Devon Primary Care Trust, Devon Partnership Trust and Torbay Care Trust. Published online: http://www.recoverydevon.co.uk/download/Recovery_Assessment_Devon-OHagan_Final.pdf (last accessed 04 June 2013).

123 Ackner B, Harris A, Oldham A. Insulin treatment of schizophrenia; a controlled study. *Lancet* 1957;**272** (6969):607–11.

